# Supplementary material for: Cerium Oxide Enhances the Toxicity of Zinc Oxide Nanoparticles in Human Lung Epithelial Cell Cultures
Source: Toxics. 2022 Sep 1;10(9):522. doi: 10.3390/toxics10090522 (PMC9502999; doi:10.3390/toxics10090522)
Supplement: Supplementary file 1 [file toxics-10-00522-s001.zip › toxics-1841623-supplementary.pdf]

Supplementary

Cerium Oxide Enhances the Toxicity of Zinc Oxide Nanoparticles in Human Lung Epithelial Cell Cultures

Tasnim Al Rashaideh[1], Nervana Metwali[2], Sarah S. Perry[2], Andrea Adamcakova-Dodd[2] and Peter S. Thorne[1,2,*]

[1]Human Toxicology Program, Graduate College, University of Iowa
[2]Department of Occupational and Environmental Health, College of Public Health, University of Iowa

*Correspondence: Peter S. Thorne: phone (319) 335-4216; peter-thorne@uiowa.edu

Calibration of sonicator

Prior to manipulating the nanomaterials, the ultrasonication apparatus must be calibrated through a calorimetric approach since each machine has a specific frequency, amplitude and delivers a certain amount of energy [1, 2]. The mean delivered acoustic power of the cup-horn sonicator was 5.4 ± 0.3 J/K.

Figure 1. shows one of the calorimetric experiments to determine the delivered power of the sonicator. The calibration was performed by sonicating a 35 mL of water in 50 mL polystyrene tube for 30 min at 75% amplitude in the presence of a recirculating chiller (Qsonica, Newtown, CT, USA. model# 4900). The change in temperature was recorded every 10 s for 30 min. Time of sonication versus the temperature of water inside the tube was plotted to calculate the delivered acoustic power p (W=J/s). The delivered acoustic power =

$$\text{slope of temperature (K)vs. time (s)} * \text{mass of water(g)} * (\text{specific heat of water4.186 (J/gK)})$$

The water level in the cup-horn sonicator must align with water level in the sample tube, to avoid variability in results. Each sonication machine delivers a specific amount of energy called the delivered sonication energy (DSE). $\text{DSE} = \text{P} * \frac{\text{t}}{\text{V}}$

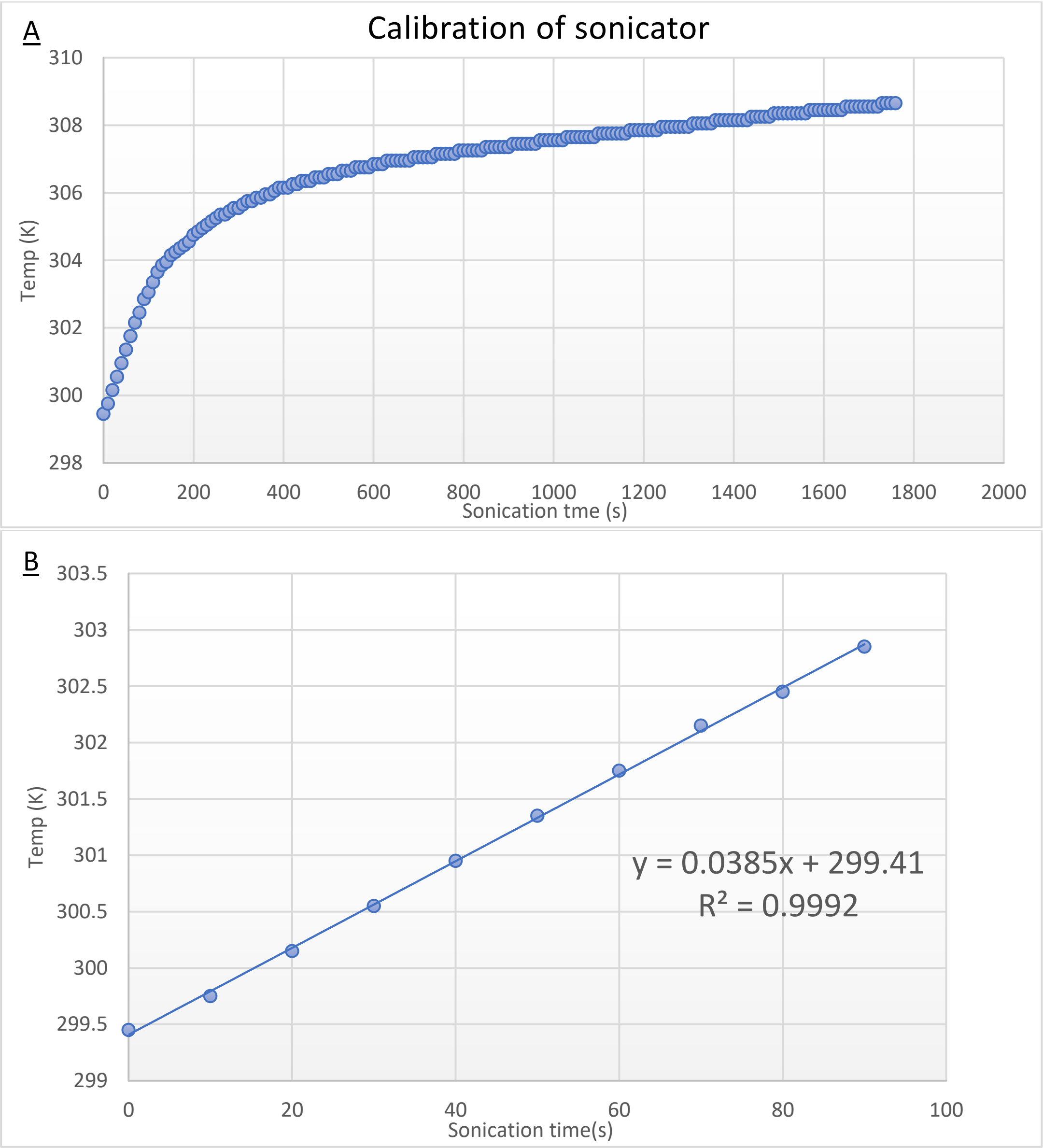

Figure S1. Calorimetric graph of sonicated water to calculate the delivered power from the sonication machine. (A)The graph shows an increase in the suspension temperature in a duration of 30 min, amplitude was 75%. (B) Represents the first 10 points of A (change of temperature in 90 sec). The delivered acoustic power= (slope from B) *mass of water *(specific heat of water 4.186 J/g K). The delivered acoustic power = 0.0385*34.88 g*4.186 J/s.

Optimum sonication duration

ENM were dispersed using a cuphorn sonicator (Model # 431C2. Qsonica, Newtown, CT). The specific duration of sonication needed for each ENM, DSE_{cr} (critical delivered sonication energy) was determined by suspending the NPs in ultrapure water (Barnstead™ GenPure™ Pro Water Purification System, ThermoFisher) to achieve the concentration of 500 μg/mL. The suspension was vortexed for 30 s then ultrasonicated for 2 min and then 1 mL of the sonicated sample was drawn and measured with the Zetasizer Nano-ZS (Malvern Instruments, Westborough, MA, USA). The drawn amount was returned to the original tube and sonicated again for 2 min. The process of sonication and measuring the hydrodynamic size continued until the ENM reached a stable minimum size.

Cumulative DSE vs. mean hydrodynamic size were plotted (**Figure 2**). According to Deloid et al., the DSE_{cr} is within 10% of the least readings, when slope equals to zero [1]. For the nanoparticles that were used in this study, further sonication for ENM after reaching the lowest hydrodynamic diameter caused the material to agglomerate as the particles started to re-agglomerate, so we picked the lowest reading we have achieved. For ZnO NPs (10 nm), the lowest size was reached after sonication for 6 min. For CeO_2 NPs (15 – 50 nm), the lowest size was achieved after 18 min of sonication.

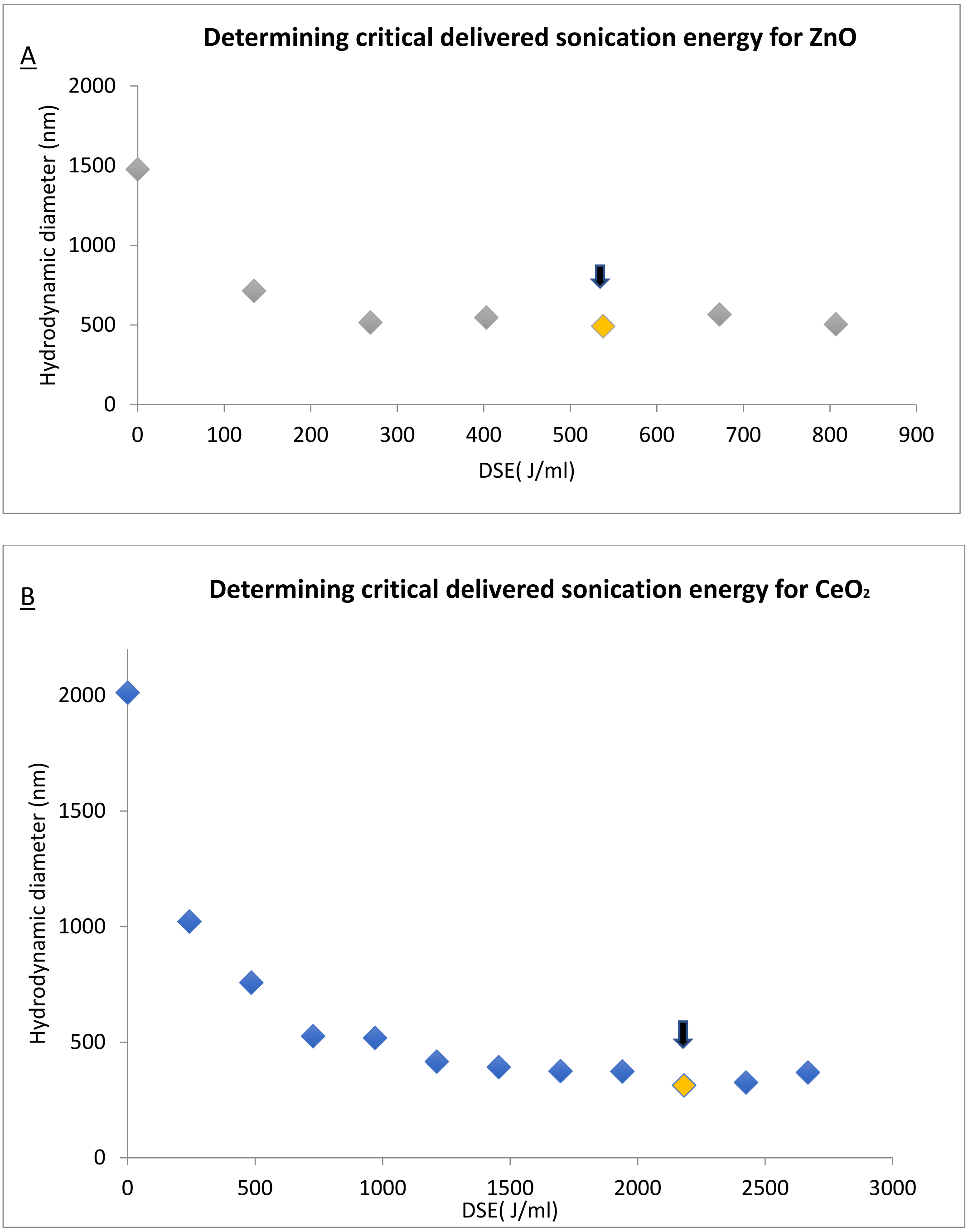

Figure S2. Determining DSEcr that produces the smallest and the most stable hydrodynamic size of NPs (A) ZnO (B) CeO_2. To calculate the optimum time for sonication for each of the NPs, we have to find the (DSEcr) that produces the smallest and the most stable hydrodynamic size. To calculate the required time for sonication t=V*DSEcr/delivered power.

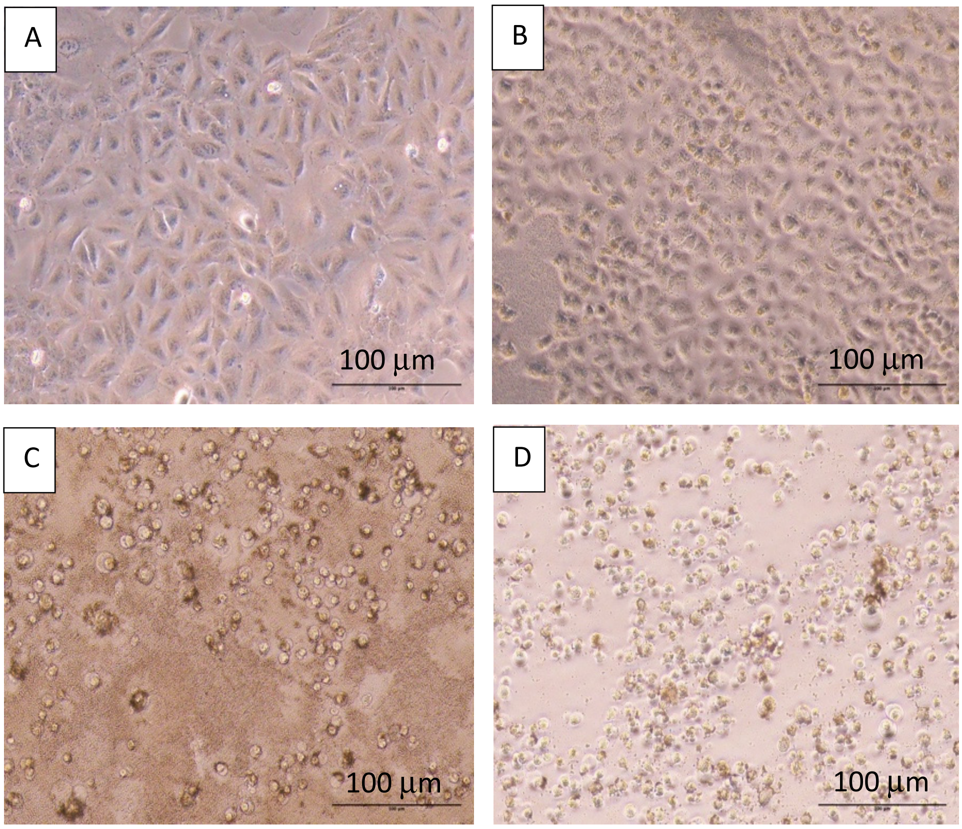

Figure S3. Microscopic images at 20x magnification of A549 cells after treatment with NPs. The normal shape (control) of A549 cells is spindle-like (A). Treatment with 62.5 µg/mL of CeO_2 had no effect on viability (B). Treatment with 62.5 µg/mL of ZnO caused loss in viability and changes in morphology from spindle shape to round shape (C). Mixture of 62.5 µg/mL of ZnO & 62.5 µg/mL of CeO_2 (D). Higher concentrations of ZnO and CeO_2 mixture reduced the number of live cells, dead cells were bright and detached from the wells to form clumps like in as shown in (D), and this agrees with viability and LDH assays. The images were obtained for the cells while in 12-well plates right after the 24hrs treatment.

Table S1. Serial dilution of NPs from stock (500 mg/mL) to prepare treatment doses. Stock concentration = 500 μg/ml, dilution factor = 2

Dilution	Volume of nanoparticles suspension added (mL)	Cell culture medium (mL) in the tube before dilution	Final concentration of nanoparticles (μg/mL)
1	8 (from 500 μg/mL stock)	8	250
2	8 (from dilution 1)	8	125
3	8 (from dilution 2)	8	62.5
4	8 (from dilution 3)	8	31.3
5	8 (from dilution 4)	8	15.6
6	8 (from dilution 5)	8	7.8
7	8 (from dilution 6)	8	3.9

Supplementary References

1. DeLoid, G. M.; Cohen, J. M.; Pyrgiotakis, G.; Demokritou, P., Preparation, characterization, and in vitro dosimetry of dispersed, engineered nanomaterials. *Nat Protoc* **2017,** *12* (2), 355-371.
2. Kaur, I.; Ellis, L. J.; Romer, I.; Tantra, R.; Carriere, M.; Allard, S.; Mayne-L'Hermite, M.; Minelli, C.; Unger, W.; Potthoff, A.; Rades, S.; Valsami-Jones, E., Dispersion of Nanomaterials in Aqueous Media: Towards Protocol Optimization. *J. Vis. Exp.* **2017,** *130*.
